# Efficacy, Safety, and Immunogenicity of Biosimilar Adalimumab Advixa® Compared With Reference Product Humira® in Patients With Rheumatoid Arthritis in Bangladesh

**DOI:** 10.7759/cureus.77638

**Published:** 2025-01-18

**Authors:** Rowsan Ara, Syed Jamil Abdal, Md. Ariful Islam, Muhammad Shoaib Momen Majumder, A T M Tanveer Hasan, Firdausi Qadri, Umme Kulsum, Sharmin Akter Mukta, Abu Bakar Siddik, Kasrina Azad, Nishat Sultana, Faez Ahmed, Md Shawkat Hossain, Minhaj Rahim Choudhury, Md. Abu Shahin, Md Nahiduzzamane Shazzad, Zannat Kawser, Syed Atiqul Haq

**Affiliations:** 1 Medicine, Green Life Medical College, Dhaka, BGD; 2 Rheumatology, Bangabandhu Sheikh Mujib Medical University, Dhaka, BGD; 3 Rheumatology, Green Life Medical College, Dhaka, BGD; 4 Biochemistry, Institute for Developing Science and Health Initiatives (ideSHi), Dhaka, BGD; 5 Biostatistics and Epidemiology, Institute for Developing Science and Health Initiatives (ideSHi), Dhaka, BGD; 6 Pharmacy, Incepta Pharmaceuticals Limited, Dhaka, BGD; 7 Genetics, Incepta Pharmaceuticals Limited, Dhaka, BGD; 8 Rheumatology, Japan Bangaladesh Friendship Hospital, Dhaka, BGD; 9 Rheumatology, Green Life Center for Rheumatic Care and Research, Dhaka, BGD

**Keywords:** adalimumab (humira), bangladesh, biosimilar, immunogenicity, rheumatoid arthiritis, safety and efficacy

## Abstract

Aim: This study evaluated the efficacy, safety, and immunogenicity of biosimilar adalimumab drug Advixa® (Incepta Pharmaceuticals Ltd, Dhaka, Bangladesh) in patients with moderate to severe rheumatoid arthritis (RA) compared to the reference drug Humira® (AbbVie Inc., North Chicago, IL, USA).

Methods: In this randomized, double-blind, prospective, parallel-group, active-controlled, non-inferiority trial, each of 144 patients was treated with six doses of Advixa® biweekly for up to 12 weeks. In the test group, there were 108 patients, and in the reference group, there were 36 patients. The primary endpoint was the proportion of the patients achieving the American College of Rheumatology 20% improvement criteria (ACR20) in response to the treatment. In contrast, ACR50, ACR70, and Disease Activity Score in 28 joints (DAS28) response were the secondary endpoints. The safety measurements included monitoring adverse events (AEs), serious AEs (SAEs), well-being assessment, and clinical laboratory abnormalities. Additionally, the level of anti-adalimumab antibody was assessed as a measure of immunogenicity against the drug.

Results: After 12 weeks, the per-protocol (PP) population treated every other week with Advixa® had statistically similar response rates as compared to Humira®: ACR20 erythrocyte sedimentation rate (ESR) (78.22% vs. 73.53%; P > 0.6), ACR50 ESR (55.45% vs. 52.94%; P > 0.8), ACR70 ESR (29.70% vs. 26.47%; P > 0.8). According to the intention to treat (ITT) population, the response rates were ACR20 (73% vs. 69%; P > 0.6), ACR50 (52% vs. 50%; P > 0.8), and ACR70 (28% vs. 25%; P > 0.8). In every criterion, the response rate for Advixa® was higher than Humira®. Similarly, the changes in DAS28-C-reactive protein (CRP) scores were -2.13 ± 1.43 vs -2.34 ± 1.55 in the PP population group (P > 0.4) and -2 ± 1.39 and -2 ± 1.56 in the ITT population group (P > 0.4) for Advixa® and Humira®, respectively. Six SAEs and 105 non-serious AEs were reported during the study. No significant difference was found between treatment groups for the incidence of SAEs (P > 0.3) and AEs (P > 0.7). There was no significant difference in the absolute value of change of anti-adalimumab antibody titer in the treatment groups from baseline to week 12 (P > 0.2).

Conclusions: The comprehensive assessment of efficacy, safety, and immunogenicity establishes the non-inferiority of Advixa® to Humira® at a 95% confidence level.

## Introduction

Rheumatoid arthritis (RA) is a debilitating autoimmune disorder characterized by chronic inflammation of the synovial joints, leading to progressive joint damage, functional impairment, and reduced quality of life [1]. The dysregulation of pro-inflammatory cytokines, including tumor necrosis factor-alpha (TNF-α), plays a pivotal role in the pathogenesis of RA [2]. Treatment options for RA include medications, lifestyle changes, supportive treatments, and surgery. Medications used to treat RA include nonsteroidal anti-inflammatory drugs (NSAIDs), corticosteroids, disease-modifying antirheumatic drugs (DMARDs), biologics, and Janus kinase (JAK) inhibitors [3]. Early treatment with certain drugs can improve long-term outcomes, and combinations of drugs may be more effective than single-drug therapy.

Targeting TNF-α has emerged as a cornerstone in managing RA, with biological therapies such as adalimumab, a fully human monoclonal antibody that binds to TNF-α, inhibiting its pro-inflammatory effects [4]. Numerous clinical trials and real-world studies have demonstrated the efficacy and safety of adalimumab in RA patients [5, 6].

Adalimumab is a human anti-TNF monoclonal antibody used to treat moderate to severe RA. Early adalimumab clinical trials in RA patients showed an excellent safety profile, with improvements in functional abilities and disease signs and symptoms, as well as the ability to establish clinical remission and stop the progression of radiographic illness [7-9]. However, the high costs of the originator molecule have limited patient access to optimized disease management and increased the cost burden for healthcare systems and individuals. The advent of biosimilars led to significant cost savings driven by price competition among the reference products, which could benefit healthcare systems [10].

This prospective, randomized, double-blind, parallel-group, active-controlled, non-inferiority study aimed to compare the efficacy, safety, and immunogenicity of the biosimilar adalimumab drug Advixa® (Incepta Pharmaceuticals Limited, Dhaka, Bangladesh) with the reference drug Humira® (AbbVie Inc., North Chicago, IL, USA).

## Materials and methods

Study design and conduction

In this randomized, double-blind, prospective, parallel-group, active-controlled, non-inferiority trial, a total of 144 patients with moderate to severe active RA were randomized in a 3:1 ratio to receive six doses of subcutaneous injections (40 mg/0.4 ml) of either Advixa® or Humira® in alternate weeks over 12 weeks. The clinical site was Green Life Center for Rheumatic Care and Research in Dhaka, Bangladesh, and the study was conducted following the principles of good clinical practice (GCP) and the declaration of Helsinki. The trial was approved by the National Research Ethics Committee (NREC) of the Bangladesh Medical Research Council (BMRC) and was registered in ClinicalTrials.gov (NCT05172817; Registration Date/Initial Release Date: 28/09/2021). The study started (patient enrollment) on 22/02/22, and the clinical phase ended on 24/02/23. Patients provided written informed consent forms before initiation of any study-related procedure.

The baseline information was recorded, and a thorough clinical examination was conducted during screening. Vital signs and laboratory examinations were taken from the patients at baseline and 12 weeks. All the injections were administered by trained study physicians at the study site. After initial screening and assessing eligibility, the randomization for this study was generated using the statistical software SAS (SAS Institute, Cary, NC, USA). The subjects were administered Advixa® or Humira® according to the randomization schedule. Allocated treatments were received by patients based on their enrollment identification number (ID) at ambient temperature in the supine position. The blinding was maintained till the end of the intervention. The patients, study investigators, and analysts concerned were blinded to the identity of the test and reference product to the individual subject.

A total of eight visits were scheduled during this study, including the screening visit (Visit 0); following the screening, enrollment Visit 1 (Day 1); Visit 2 (Day 15 ± 1); Visit 3 (Day 28 ± 3); Visit 4 (Day 42 ± 3); Visit 5 (Day 56 ± 3); Visit 6 (Day 70 ± 3); and Visit 7 (Day 84 ± 3). The study drugs were administered on Visit 1 through Visit 6. Physical examinations were done on each visit, including vital signs measurement and well-being assessment. Hematological tests (complete blood count (CBC), C-reactive protein (CRP), and erythrocyte sedimentation rate (ESR)) and important biochemical parameters, including serum glutamic pyruvic transaminase (SGPT) and serum creatinine, were evaluated during screening and on Visits 1, 3, 5, and 7.

Study participants

We recruited RA patients according to the 2010 American College of Rheumatology (ACR)/European League Against Rheumatism (EULAR) classification criteria. An inadequate response to treatment was defined as failure to achieve a 50% reduction in the Disease Activity Score in 28 joints (DAS28) with 15 to 20 mg of methotrexate (MTX) at the end of 12 weeks or failure to achieve at least a low disease activity (DAS28 <3.2) with oral or subcutaneous MTX 20-25 mg per week for at least eight weeks within a period of its use for six months before screening. Adult human RA patients in the range of 18-65 years (both inclusive) with an inadequate response to MTX and providing informed written consent were considered for enrollment. The subjects were evaluated for eligibility to participate in the study based on the inclusion and exclusion criteria, demographic characteristics, physical examination including vital signs, 12-lead ECG reports, chest X-ray reports (when required), and laboratory reports. Other exclusion criteria included cytopenias, renal or hepatic impairment, pregnancy or desire for pregnancy, lactation, and the presence of current active infections, including tuberculosis (TB), and malignancies. The patients with latent TB infection (LTBI) were put on LTBI treatment according to CDC guidelines (preferably three months of daily rifampicin and isoniazid combination).

Efficacy assessment

**Figure 1:**
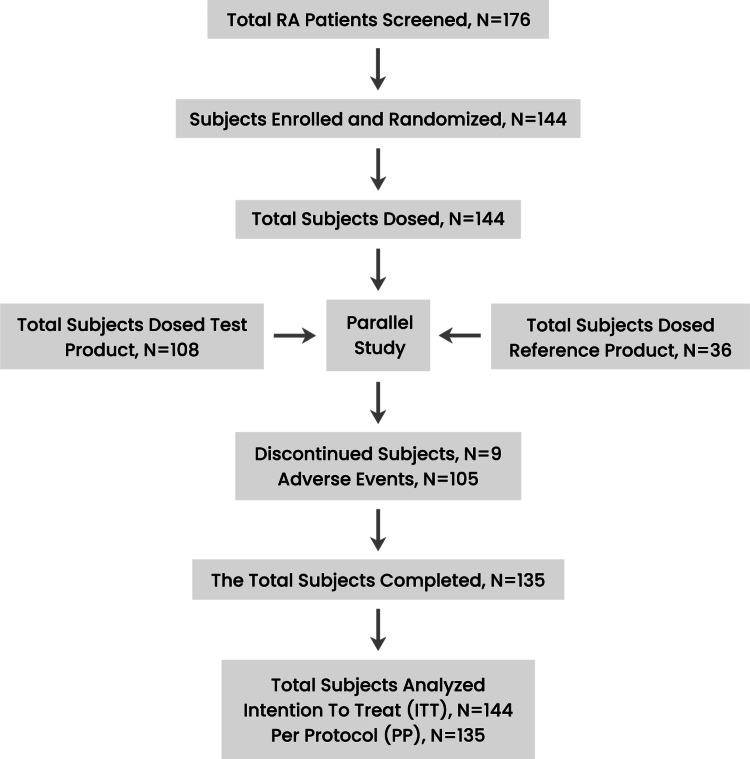
Disposition of study subjects RA: rheumatoid arthritis

As shown in Figure 1, of the 176 screened participants, 32 were excluded due to their meeting the exclusion criteria, mostly active tuberculosis and other active systemic infections. Several scores and composite outcomes, were used in this clinical trial to evaluate comparable efficacy between the test and reference products among the intention to treat (ITT) (N=144) and per-protocol (PP) (N=135) populations. The primary efficacy outcome was determined based on the proportion of patients with an American College of Rheumatology 20% improvement criteria (ACR20) response in both treatment groups at week 12. The secondary efficacy outcomes were the DAS28‐CRP, the proportion of patients with ACR50 response, and ACR70 response in both treatment groups at week 12.

Safety assessment

A physical examination, including vital signs and well-being assessment, was done during each visit. Biochemistry and hematology tests were conducted to assess the safety of the products, and the results were compared at Day 84 post dose or on discontinuation of the subject from the study. Safety measurements included monitoring adverse events (AEs), serious AEs (SAEs), physical examination, vital signs, and clinical laboratory results. After each dosage administration, active/solicited safety follow-up for AEs was performed for two days, and passive/unsolicited safety follow-up was performed for the remaining 11 days. This monitoring was repeated after each dosage up to the completion of the study.

Immunogenicity assessment

The level of anti-adalimumab antibody was determined in 116 randomly selected samples as a measure of immunogenicity at baseline and within four weeks after the last injection with the study agents (Week 12). Serum concentrations of antibodies for adalimumab were measured using adalimumab (Humira®) anti-drug antibody enzyme-linked immunosorbent assay (ELISA) kits (Somru BioScience, Charlottetown, Canada) following the manufacturer’s instructions. Positive and negative ELISA results were calculated based on the pre-determined cutoff value of 0.026.

Statistical analysis

With an allocation ratio of three, the sample size of 35 participants in the Humira® arm and 105 participants in the Advixa® arm was estimated by a non-inferiority margin of δ=−0.15 that was based on clinical judgment with 80% power and a 5% level of significance. Primary efficacy measures were evaluated using ITT and PP populations, while the ITT population was used for safety assessment. Data were analyzed using R software version 4.2.3 (The R Core Team, R Foundation for Statistical Computing, Vienna, Austria). P-values were calculated using the chi-square, Pearson’s chi-square, Fisher’s exact, and Mann-Whitney-Wilcoxon tests. A value <0.05 was considered significant. Descriptive analysis and tables were prepared for better representation.

## Results

One hundred forty-four subjects who met the inclusion criteria were enrolled after screening 176 participants from February to November 2022. Table 1 displays the baseline information of the participants subjected to the randomization for the investigational product (IP) test group Advixa® and reference group Humira® with a ratio of 3:1. Patients from both arms had comparable baseline characteristics, resulting in a non-significant difference between the two groups during the selection process.

**Table 1:** Baseline demographic and clinical characteristics of the study subjects (N=144) Data are presented as n (%) and median (IQR). *P-value was calculated using Fisher’s exact test for categorical values. P-values were calculated using the Wilcoxon rank sum test for quantitative non-parametric values. IQR: interquartile range

Variables	Adalimumab (reference; N=36)	Adalimumab (test; N=108)	p-value
Gender n (%)			
Male	5(14%)	10(9%)	0.63*
Female	31(86%)	98(91%)	
Age (years)			
Median (IQR)	44 (38, 53)	48 (39, 55)	0.276
Body mass index (kg/m^2^)			
Median	27.1 (23.3, 30.8)	27.0 (23.2, 28.9)	0.523
Duration of the disease (years)			
Median (IQR)	3 (2, 5)	3 (2, 4)	0.976
White blood cells (WBC) (×10^9/L)			
Median (IQR)	8.00 (6.72, 9.85)	8.25 (7.00, 10.00)	0.53
Hemoglobin (g/dl)			
Median (IQR)	10.15 (9.53, 11.00)	11.00 (9.70, 11.40)	0.055
Platelet count (PLT) (×10^9/L)			
Median (IQR)	316 (260, 385)	330 (268, 400)	0.681
Serum creatinine (mg/dl)			
Median (IQR)	0.90 (0.80, 1.07)	0.87 (0.80, 1.00)	0.371
Erythrocyte sedimentation rate (ESR) (mm/hr)			
Median (IQR)	57 (37, 73)	57 (38, 74)	0.972

Efficacy analysis

The proportions of patients achieving ACR20, ACR50, or ACR70 at baseline and week 12 following treatment with Advixa® or Humira® are shown in Table 2. Per protocol, efficacy analysis was performed on 135 subjects (Table 2), where nine were excluded because of missed follow-ups at different time points, resulting in subject dropout. Efficacy analysis in the ITT population was done on 144 subjects (Table 3), considering no change of efficacy parameters from baseline till week 12 for the nine dropout subjects.

**Table 2:** Adalimumab efficacy analysis: primary efficacy outcome (ACR20 response) and secondary efficacy outcome (ACR50 and ACR70 response) in the PP population (N=135) P-values were calculated from Pearson’s chi-squared test. ACR: American College of Rheumatology; ESR: erythrocyte sedimentation rate; CRP: C- reactive protein; PP: per-protocol

	Humira® (reference; N=34)		Advixa® (test; N=101)		Chi-squared	95% CI		P-value
	Response	%	Response	%	Test statistic	Lower (%)	Upper (%)	
ACR20 (ESR)	25	73.53	79	78.22	0.11	0.29	2.17	0.744
ACR20 (CRP)	25	73.53	78	77.23	0.04	0.31	2.29	0.837
ACR50 (ESR)	18	52.94	56	55.45	0.00	0.38	2.13	0.956
ACR50 (CRP)	18	52.94	57	56.44	0.02	0.37	2.05	0.876
ACR70 (ESR)	9	26.47	30	29.70	0.02	0.31	2.17	0.887
ACR70 (CRP)	9	26.47	31	30.69	0.06	0.29	2.07	0.803

**Table 3:** Adalimumab efficacy analysis: primary efficacy outcome (ACR20 response) and secondary efficacy outcome (ACR50 and ACR70 response) in the ITT population (N=144) P-values are calculated from Pearson’s chi-squared test. ACR: American College of Rheumatology; ESR: erythrocyte sedimentation rate; CRP: C-reactive protein; ITT: intention to treat

	Humira® (reference; N=36)		Advixa® (test; N=108)		Chi-squared	95% CI		P-value
	Response	%	Response	%	Test statistic	Lower (%)	Upper (%)	
ACR20 (ESR)	25	69.44	79	73.15	0.05	0.29	2.17	0.83
ACR20 (CRP)	25	69.44	78	72.22	0.01	0.31	2.29	0.91
ACR50 (ESR)	18	50.00	56	51.85	0.00	0.38	2.13	0.99
ACR50 (CRP)	18	50.00	57	52.78	0.01	0.37	2.05	0.92
ACR70 (ESR)	9	25.00	30	27.78	0.01	0.31	2.17	0.91
ACR70 (CRP)	9	25.00	31	28.70	0.05	0.29	2.07	0.82

According to the PP population, after 12 weeks, patients treated every other week with Advixa® had statistically similar response rates as compared to Humira® which were as follows: ACR20 ESR (78.22% vs. 73.53%), ACR20 CRP (77.23% vs. 73.53%), ACR50 ESR (55.45% vs. 52.94%), ACR50 CRP (56.44% vs. 52.94%), ACR70 ESR (29.70% vs. 26.47%), ACR70 CRP (30.69% vs. 26.74%).

P-values were calculated from Pearson’s chi-squared test.

Like the PP population, the efficacy analysis of the ITT population, after 12 weeks, showed that patients treated with Advixa® had statistically similar response rates as compared to Humira® which were as follows: ACR20 ESR (73.15% vs. 69.44%), ACR20 CRP (72.22% vs. 69.44%), ACR50 ESR (51.85% vs. 50.00%), ACR50 CRP (52.78% vs. 50.00%), ACR70 ESR (27.78% vs. 25.00%), ACR70 CRP (28.70% vs. 25.00%). In every criterion, the response rate for Advixa® was higher than Humira® in both PP and ITT populations.

The other two secondary efficacy outcomes (DAS28-ESR and DAS28-CRP) are shown in Table 4 and Table 5 at week 12 of IP administration for both the PP and ITT populations. The absolute values of change in the DAS28-ESR and DAS28-CRP scores were not significantly different between the two products. The proportion of 50% improvement in the DAS28-ESR score was the same between Advixa® and Humira® groups, while it was higher in the test group for the DAS28-CRP score.

**Table 4:** Adalimumab efficacy analysis: secondary efficacy outcomes (DAS-28 ESR and DAS-28 CRP) in the PP population Data presented as mean ± SD and n (%); P-values were calculated using *Mann-Whitney-U Test, **Student’s t-test, and *** Pearson’s chi-squared test. DAS-28: Disease Activity Score in 28 joints; ESR: erythrocyte sedimentation rate; CRP: C-reactive protein; PP: per protocol

	Humira® (reference; N=34)	Advixa® (test; N=101)	Test statistics	P-value
DAS-28 (ESR)				
Baseline	7.05 ± 0.85	6.8 ± 1.03	2055	0.161*
Week 12	4.89 ± 1.59	4.63 ± 1.49	0.84	0.41**
50% improvement	6 (16.67%)	18 (16.67%)	0	1.000***
Absolute value of change from baseline	-2.15 ± 1.52	-2.16 ± 1.33	0.03	0.985**
DAS-28 (CRP)				
Baseline	6.27 ± 0.80	5.91 ± 1.02	2248	0.038*
Week 12	3.93 ± 1.52	3.77 ± 1.45	0.54	0.611**
50% improvement	7 (19.44%)	28 (25.92%)	0.67	0.551***
Absolute value of change from baseline	-2.34 ± 1.55	-2.13 ± 1.43	-0.7	0.494**

**Table 5:** Adalimumab efficacy analysis: secondary efficacy outcome (DAS-28 ESR and DAS-28 CRP) in the ITT population Data presented as mean ± SD and n (%); P-values were calculated using *Mann-Whitney-U Test, **Student’s t-test, and *** Pearson’s chi-squared test. DAS-28: Disease Activity Score in 28 joints; ESR: erythrocyte sedimentation rate; CRP: C-reactive protein; ITT: intention to treat

	Humira® (reference; N=36)		Advixa® (test; N=108)	Test statistics	P-value
DAS-28 (ESR)					
Baseline	6.65 ± 1.83		6.35 ± 1.95	2250	0.195*
Week 12	4.61 ± 1.91		4.33 ± 1.83	0.77	0.436**
50% improvement	6 (16.66%)		18 (16.66%)	0	1.000***
Absolute value of change from baseline	-2 ± 1.56		-2 ± 1.39	0	1.000**
DAS-28 (CRP)					
Baseline	5.91 ± 1.64		5.52 ± 1.76	2341	0.119*
Week 12	3.7 ± 1.73		3.52 ± 1.68		0.591**
50% improvement	7 (19.44%)		28 (25.92%)	0.31	0.574***
Absolute value of change from baseline	-2.2 ± 1.59		-1.9 ± 1.47	-0.998	0.478**

Safety analysis

Overall, the incidence of AEs was comparable between patients who received Advixa® and those who took Humira® (Table 6).

**Table 6:** Summary of information relating to the adverse events in the treatment groups Data are presented in the form of n (%); *P-values were calculated from Pearson’s chi-square test; **P-values are calculated from Fisher’s exact test

Adverse events	Humira® (reference; N=36)	Advixa® (test; N=108)	Chi-squared test statistics	P-value
Non-serious adverse events	18 (50.0%)	59 (54.6%)	0.43	0.52*
Serious adverse events	0 (0.0%)	5 (4.6%)	1.92	0.3315**

One hundred five non-serious AEs and six SAEs were recorded during the 12-week treatment schedule of six doses of adalimumab. Within 105 non-serious AEs, 79 AEs were reported by 59 (54.6%) subjects receiving Advixa®, and 26 AEs were reported by 18 (50%) subjects receiving Humira®. Complaints of dizziness, weakness, increased frequency of loose stool, headache, palpitation, abdominal cramp, anorexia, nausea, itching over the site of IP administration, cough, hair loss, generalized itching with rash, vomiting, and glossitis with gingivitis were among the most frequently reported non-serious AEs during the entire course of the study. The frequency distribution of non-serious AEs by Humira® and Advixa® is represented in Appendix A. The reported AEs were mild in severity, unlikely related to the study medication, and resolved spontaneously or with treatment. There was no significant difference in the incidence of non-serious AEs between the two treatment groups at a 95% confidence level as recorded during the study time frame, with all P-values > 0.05.

A total of six serious adverse events were reported during the entire study. One death (diagnosed as urinary tract infection with impending septic shock) and five conditions requiring hospitalization (ischemic stroke with electrolyte imbalance, road traffic accident (RTA) followed by transient memory loss, dengue with COVID-19, acute gastroenteritis, and severe chest pain due to pneumonitis). During analysis, RTA was excluded from the dataset due to the absence of any causal relationship. All the SAEs were reported from participants receiving test drugs. However, there was no significant difference in the occurrence of SAEs (P-value = 0.33). A similar non-significant distribution was noted for non-serious AEs (P-value = 0.52) between the test (n = 59, 54.6%) and reference product (n = 18, 50%).

Immunogenicity analysis

No significant difference was found in the titer of the anti-adalimumab antibody (P-value = 0.89) and the absolute change in the value from baseline to week 12 (P-value = 0.26) between the treatment groups. Low-levels of anti-drug antibody were observed in two baseline samples. No apparent correlation of antibody development to adverse reactions was observed.

## Discussion

A biosimilar must be like the reference biological product regarding structural and functional assays, pharmacokinetics, efficacy, safety, and immunogenicity to be considered potential [11]. Adalimumab is associated with a rapid clinical response rate following subcutaneous injection, providing similar benefits at 40 and 80 mg doses. Our study was designed to evaluate the efficacy, safety, and immunogenicity of test adalimumab Advixa® in comparison with the reference drug Humira® treatment in patients with moderate to severe RA at fortnightly subcutaneous dosing of 40 mg, following the statement of international consensus on biological agents. The present study was conducted after completing the pharmacokinetic and safety analysis of the concerned biosimilar adalimumab in healthy volunteers in Bangladesh, demonstrating pharmacokinetic bioequivalence between Advixa® and Humira® [12]. The sample size was estimated with the help of expert medical knowledge before the regulatory approvals and the study initiation.

Several studies assessed the efficacy of 40 mg adalimumab administered subcutaneously every other week regarding ACR criteria and DAS-28 response [13, 14]. Our results demonstrated equivalent efficacy between the Advixa® and Humira® about the primary endpoint (ACR20 response) and secondary efficacy endpoints (ACR50, ACR70, and DAS28). More than 70% of the ACR20 response rate observed in this study was comparable to that demonstrated in other studies of adalimumab [15], with some reporting higher responses [16, 17]. In comparison, some reported a lower response than the current study [18,19]. Regarding the secondary efficacy parameters, data are available at Week 12, Week 24, and beyond [20, 21]. In the study by Fleischmann et al., the efficacy of reference adalimumab after 26 weeks of treatment was assessed in RA patients, where 52% (~40% at Week 12) of patients reported ACR50 response and 28% (~20% at Week 12) reported ACR20 response [15]. Very close observations as ours for changes of absolute values of DAS28-ESR (2.95±1.3) and DAS28-CRP (2.1±1.09) scores were reported in previous studies [16,17].

Most AEs were mild and resolved spontaneously or with treatment. The distribution of adverse events was comparable between the treatment groups. The reported AE rates of Advixa® and Humira® groups were like those of treatment-emergent AEs in previous studies [20]. The spectrum of AEs we noticed was consistent with those reported in other studies [21]. In our study, injection site reactions were not evident in any treatment arms, which is a commonly reported AE with adalimumab [16].

A total of six SAEs were reported during the entire course of the study. Four recovered, one dropped out but recovered, and one resulted in death. The subject who encountered death was hospitalized due to abdominal pain, diarrhea, and fever seven days after receiving two doses of Advixa®. Despite intensive care unit (ICU) management, the patient died after eight days of ICU stay due to neutropenic sepsis with pneumonia, septic cardiomyopathy, and shock. A causality assessment was done, and the SAE was considered possibly related to the study medication. However, MTX-induced bone marrow suppression was assumed to be the cause of death upon investigation. Regarding the causality relationship, ‘possible’ is defined as a clinical event, including laboratory test abnormality, with a reasonable time sequence to administering the drug, but which could also be explained by concurrent disease or other drugs or chemicals (The World Health Organization-Uppsala Monitoring Centre (WHO-UMC) Causality Assessment). The observed SAEs in this study were consistent with reported cases [22].

The immunogenicity assessment was performed in a subsample of the study population at week 12, and the study was not powered to assess immunogenicity. In both arms, the incidence of binding ADA positivity increased without having any significant difference. However, the rates were lower [23] and similar [16] compared to the findings in other studies. The data reflecting the percentage of patients whose test results are considered positive for ADA in an ELISA assay are highly dependent on the sensitivity and specificity of the ELISA assay. Additionally, several key factors, including sample handling, timing of sample collection, concomitant medications, and underlying disease, may influence the observed positivity rates in an assay [21].

Limitations

Immunogenicity assessment could not be done in 100% of samples. To establish immunogenic biosimilarity, further assessment of the correlation between the neutralizing antibody and clinical improvement in RA patients regarding ACR response needs to be done.

## Conclusions

According to the study, the reference product Humira® and the test product adalimumab Advixa® both showed similar efficacy in treating RA. There were no appreciable variations in adverse events or side effects between the two medicines, which had comparable tolerance and safety profiles. In addition, it was found that Advixa® and Humira® had equivalent immunogenicity, meaning that patients’ immune responses to both medications were identical.

## Appendices

Appendix A 

**Table 7:** Frequency of non-serious adverse events by reference product and test product

Complaints	Frequency	By Humira® (reference product)	By Advixa® (test product)
Dizziness	13	2	11
Increased frequency of loose stool	9	4	5
Weakness	9	4	5
Headache	7	0	7
Palpitation	7	3	4
Abdominal cramp	7	0	7
Itching over the site of investigational product (IP) administration	3	1	2
Cough	6	2	4
Hair loss	5	2	3
Anorexia	5	2	3
Nausea	3	0	3
Vomiting	6	2	4
Generalized itching with rash	9	1	8
Glossitis with gingivitis	3	1	2
Constipation	1	0	1
Active fungal infection	3	1	2
Abscess	2	0	2
Menorrhagia	1	0	1
Hypopigmentation	1	0	1
Generalized burning sensation	2	0	2
Cervical lymphadenopathy	1	1	0
Bodyache and pitting edema on legs	1	0	1
Black spots over legs, feet, and hand	1	0	1
Total non-serious adverse events	105	26	79
